# Preserving Essential Skills: The Future of Vaginal Hysterectomy Training in Urogynaecology

**DOI:** 10.1111/1471-0528.17974

**Published:** 2024-10-02

**Authors:** Reut Rotem, Michael O. Carey, Claire M. McCarthy, Barry A. O'Reilly, Yair Daykan, Orfhlaith E. O'Sullivan

**Affiliations:** ^1^ Department of Urogynaecology Cork University Maternity Hospital Cork Ireland; ^2^ Department of Obstetrics & Gynecology, Shaare Zedek Medical Center, Faculty of Medicine Hebrew University School of Medicine Jerusalem Israel; ^3^ Department of Obstetrics and Gynaecology University College Dublin Dublin Ireland; ^4^ The Department of Obstetrics and Gynecology Meir Medical Center Kfar Saba Israel; ^5^ School of Medicine, Faculty of Medical and Health Sciences Tel‐Aviv University Tel‐Aviv Israel

**Keywords:** subspecialisation, surgical training, surgical volume, training requirements, vaginal hysterectomy

## Abstract

**Objectives:**

This study aimed to evaluate the training and self‐assessed proficiency of surgeons in the surgical management of pelvic organ prolapse (POP). We focused on the factors that influence decision‐making, the surgical techniques employed, the training received, and the management of complications.

**Design:**

A cross‐sectional survey.

**Setting:**

An electronic questionnaire.

**Population:**

European Urogynaecological Association (EUGA) and International Urogynecological Association (IUGA) members.

**Methods:**

A total of 33 questions evaluating surgeon preference regarding vaginal surgeries.

**Main Outcome Measures:**

Demographics, surgical selection, proficiency and technique, and training methods.

**Results:**

There were 471 respondents, of which 273 (58%) dedicated more than 50% of their week to urogynaecology. 250 (53%) had completed a fellowship, with 215 (86%) of those fellowships being in urogynaecology and pelvic floor reconstruction. A preference for hysterectomy in cases of uterine descent was noted by 297 (63%) respondents, influenced mainly by patient preference, age, and prolapse anatomical score. A total of 443 (94%) were proficient in vaginal hysterectomy, with two‐thirds performing 30 or fewer procedures annually; 212 (45%) reporting a decrease in the number of procedures over the last decade. Additionally, 373 (79%) respondents believed that 10–30 cases were needed to achieve and maintain proficiency.

**Conclusion:**

Vaginal hysterectomy remains a key component in uterine prolapse repair. However, with the rise of uterine‐sparing prolapse repairs, the decision‐making process may be influenced by multiple factors, including surgical training. Emphasis should be placed on training and maintaining proficiency in both traditional and novel techniques.

## 1 Introduction

Pelvic organ prolapse (POP) is a prevalent condition affecting a significant proportion of the global female population; estimates suggest that it impacts up to 50% of women over the age of 50 [1]. POP can be managed conservatively or surgically, depending on the severity and patient preference [2]. In the surgical management of uterine prolapse, it is well acknowledged that apical suspension is crucial for a successful outcome [3]. In recent years, there has been a trend towards more minimally invasive procedures, particularly those that preserve the uterus [4]. These techniques, including sacrohysteropexy, high uterosacral ligament suspension, and sacrospinous ligament fixation, have demonstrated similar functional outcomes to traditional hysterectomy‐based approaches [5, 6]. This shift in practice is well documented in the United States [7] where from 2002 to 2010 the number of hysterectomies performed annually declined significantly from 681 234 in 2002 to 433 621 in 2010. The use of vaginal hysterectomy has also decreased since 1998, from 24.8% of all hysterectomies in 1998 to 16.7% in 2010 [7]. Specifically, hysterectomies for POP decreased from 122 495 cases in 2002 to 74 230 procedures in 2010 [7]. Nevertheless, vaginal hysterectomy remains the safest operative approach compared to both abdominal and minimally invasive procedures [8]. In many cases in which the uterus must be removed for other indications, such as menorrhagia in the presence of POP, vaginal hysterectomy is the most appropriate choice.

The trend of abandoning vaginal hysterectomy in favour of other procedures can diminish a surgeons' repertoire owing to reduced case numbers of this essential and important surgical technique [9]. Maintaining proficiency in surgical techniques requires regular practice [10]. A systematic review and meta‐analysis indicated that low‐volume gynaecological surgeons had a 40% higher risk of any in‐hospital complication, an 80% higher risk of intraoperative complications, and a 50% higher risk of postoperative complications compared to high‐volume surgeons [10]. This underscores the critical need for consistent and comprehensive surgical training.

Given the shift towards minimally invasive and uterine‐sparing procedures, there is a growing concern that vaginal hysterectomy—a procedure long considered a cornerstone in the management of POP—may be at risk of becoming obsolete [11]. This potential “death” of vaginal hysterectomy has significant implications not only for patient outcomes but also for the training of future generations of gynaecologists [11].

The aim of this study is to assess surgical training and proficiency concerning various techniques for uterine prolapse, with a particular focus on the status and future of vaginal hysterectomy. Specifically, we aim to identify the factors influencing the choice of surgical route, decision‐making processes, and the management of complications. This study seeks to shed light on whether vaginal hysterectomy is at risk of becoming a lost procedure and how this might affect the training and proficiency of future generations of surgeons.

## 2 Methods

### 2.1 Study Design and Participants

A cross‐sectional survey was conducted in 2019. The survey was distributed by the International Urogynecological Association (IUGA) and the European Urogynaecological Association (EUGA), with a combined membership estimated to be over 3000 individuals [12]. The non‐validated questionnaire was developed by two board‐certified urogynaecologists with accredited sub‐specialty programs (BO, OOS). While the use of a non‐validated questionnaire introduces potential limitations to the study—such as the possibility of measurement bias and a reduced ability to generalise the findings—it is important to note that the questionnaire was meticulously designed to comprehensively cover relevant topics in the field. Additionally, it was reviewed, commented on, and approved by the research and development committees of both IUGA and EUGA.

### 2.2 Survey Instrument

The questionnaire consisted of 33 questions (Appendix S1). The initial questions addressed demographics and professional background, followed by surgical preferences and factors influencing the choice of uterine removal versus preservation. The survey then covered proficiency and frequency of procedures, concluding with training and competency. Specific attention was given to vaginal hysterectomy, including the number performed, perceived difficulty, management of intraoperative complications, and concomitant surgeries.

### 2.3 Data Collection

The questionnaire was emailed to all members of IUGA and EUGA, and data were collected using an online survey platform. This distribution was conducted in accordance with the regulations of the IUGA and EUGA executive boards, and proper Helsinki approval was waived accordingly. All responses were anonymised to ensure confidentiality. No funding was received for the development or distribution of the questionnaire, nor for the data analysis.

### 2.4 Data Analysis

The completed data forms were analysed using SPSS (Version 27; IBM, NYC, USA). Descriptive statistics were used to summarise the demographic data, surgical preferences, proficiency, frequency of procedures, and training aspects. Out of the 471 participants, response rates for individual questions varied, as respondents were not required to answer all questions. To address this, each question is presented with the denominator indicating the percentage of the total who answered.

## 3 Results

### 3.1 Demographics and Professional Background

There were 471 respondents (Table 1). The majority were urogynaecologists (70%) and obstetricians/gynaecologists (19%). Predominantly, they were consultants (71%) working in university or tertiary hospitals (56%). Additionally, 74% had over 10 years of practice, with 37% having more than 20 years.

**TABLE 1:** Respondent demographics.

Category	Count (%)
Total respondents	471 (100)
Specialty	
Urogynaecologists	328 (70)
Obstetricians and gynaecologists	90 (19)
Gynaecologists	47 (10)
Urologists	6 (1)
Level of practice	
Consultants	334 (71)
Staff grade level	80 (17)
Practice location	
University/tertiary hospitals	259 (56)
District/private hospitals	160 (34)
Years in practice	
More than 10 years	346 (74)
More than 20 years	173 (37)

When questioned about the amount of time dedicated to urogynaecology, 273 (58%) respondents spent more than 50% of their working week exclusively on urogynaecology, while 24 (5%) spent less than 10% of their time in this field. A total of 251 (53%) had completed a fellowship, with the majority (215, 86%) in urogynaecology and pelvic floor reconstruction. Furthermore, 215 (86%) of these fellowships were between 1 and 3 years in duration. Specifically, 99 respondents (39%) completed their fellowship in Europe, and a further 59 (23%) undertook their fellowship in the USA.

### 3.2 Surgical Practices and Influencing Factors

In total, 297 (63%) respondents highlighted a preference for uterine removal in the presence of uterine descent. The main factors influencing decisions were patient preference (362, 77%), patient age (259, 55%), and prolapse score (198, 42%). Additionally, 118 (25%) indicated that surgical training impacted their decision‐making regarding hysterectomy, while 157 (33%) were influenced by recurrence rates, which varied across different surgical methods.

### 3.3 Proficiency and Frequency of Procedures

**FIGURE 1:**
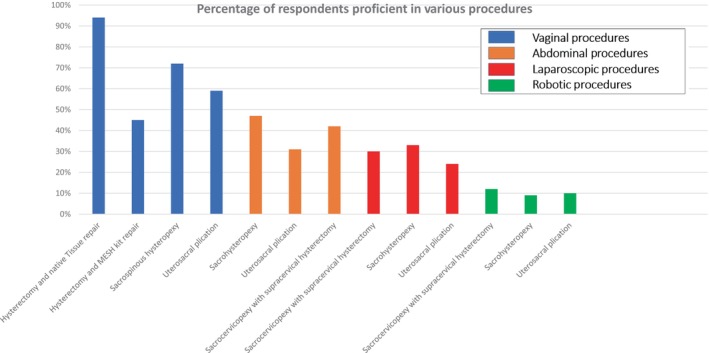
Percentage of respondents proficient in various procedures.

**FIGURE 2:**
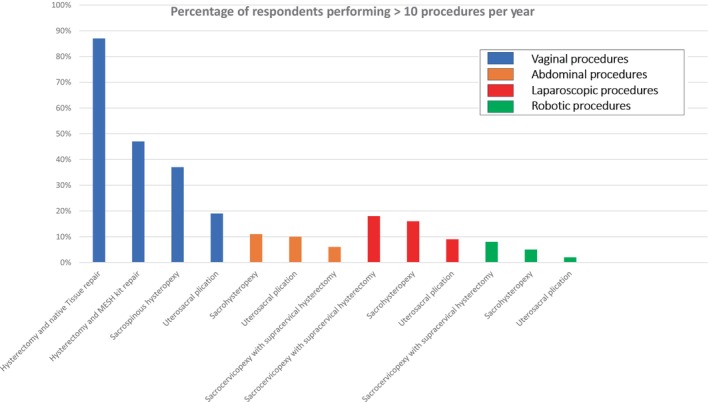
Percentage of respondents performing > 10 procedures per year.

Self‐proficiency in performing vaginal hysterectomy and repair was reported by the majority (443, 94%) of respondents. The proficiency and number of procedures performed per year for other surgical procedures varied widely (Figures 1 and 2, respectively). The procedure with the highest proficiency was vaginal hysterectomy and native tissue repair (442, 94%), followed by vaginal uterosacral plication (279, 59%). The least proficient procedure was robot‐assisted uterosacral plication (44, 9%). The most frequently performed procedure, done more than 10 times a year, was vaginal hysterectomy and native tissue repair (410). Moreover, two‐thirds of respondents (314, 66.6%) perform 30 or fewer vaginal hysterectomies per year, with 212 (45%) noting a reduction in the number compared to 5–10 years ago. Furthermore, 212 (45%) never perform a concomitant bilateral salpingo‐oophorectomy (BSO), while 10 (2%) always do. A total of 52 (11%) clamp the uterosacrals extraperitoneally, and 434 (92%) always perform a suspensory suture at the time of vaginal hysterectomy. Of those performing suspensory sutures, 190 (40%) use the McCall culdoplasty or a modification of it, while 105 (22%) perform a concomitant sacrospinous fixation. When performing a vaginal hysterectomy, 434 (92%) will do a native tissue repair, while the remaining 37 (8%) will use either a synthetic or animal graft. Additionally, 71 (15%) routinely perform an anti‐incontinence procedure, with the majority (62, 88%) using a mid‐urethral sling. Moreover, 324 (69%) do not routinely perform cystoscopy at the time of surgery.

Regarding the management of intraoperative complications, 405 (86%) would close a bladder injury defect themselves and continue the surgery, while 259 (55%) would close a rectal injury defect and continue the surgery, and 160 (34%) would call colorectal surgeons to close the defect and then continue the procedure.

### 3.4 Training and Competency

**FIGURE 3:**
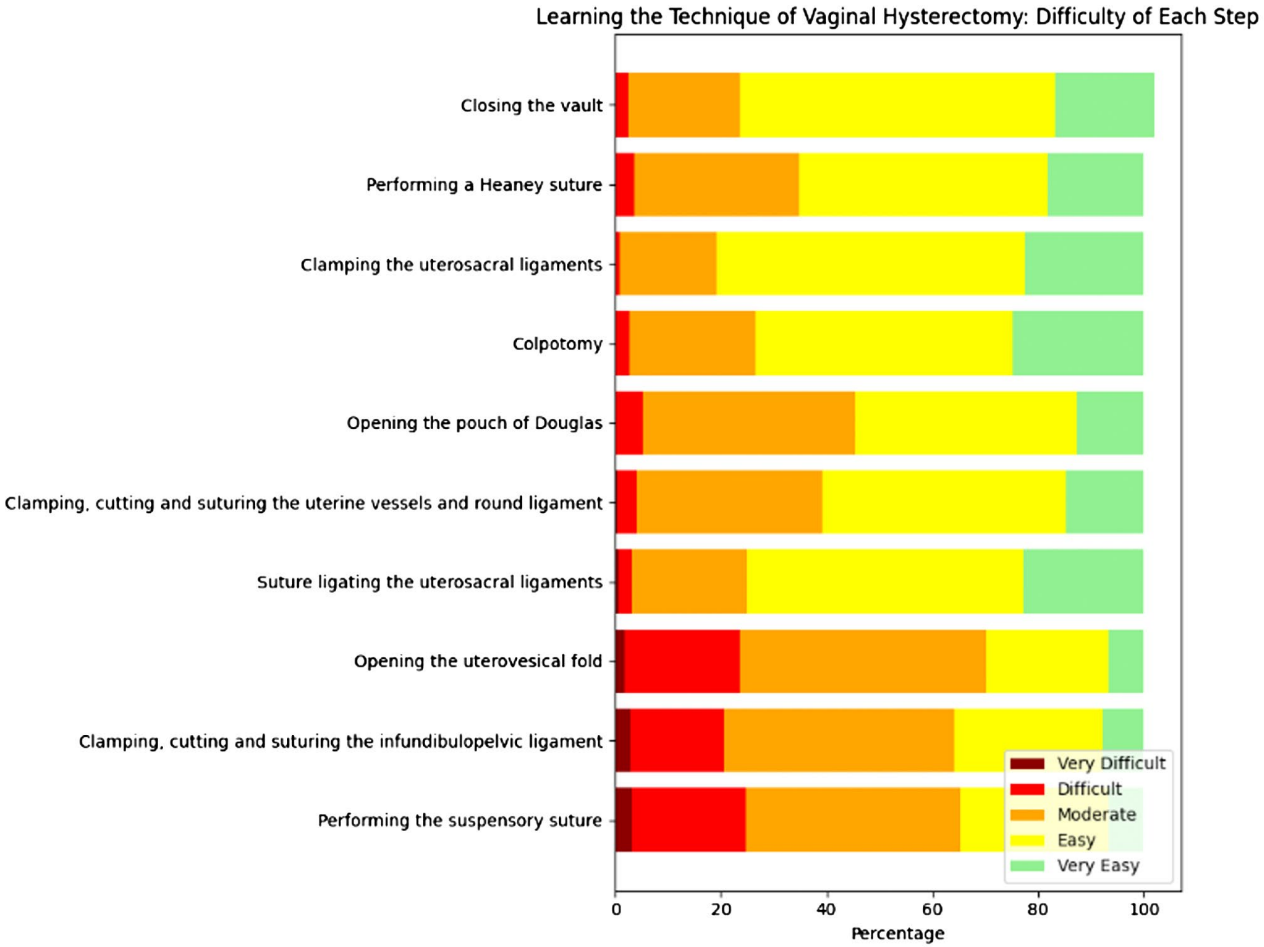
Learning the technique of vaginal hysterectomy: Difficulty of each step.

With specific regard to training and learning to perform a vaginal hysterectomy, respondents were asked to grade the complexity of each step from very easy to very difficult (Figure 3). The most difficult step to learn was perceived to be opening the uterovesical fold (103, 21.9%), followed by performing the suspensory suture (100, 21.5%). The easiest step to learn was colpotomy (116, 24.9%), followed by suture ligation of the uterosacral ligament (107, 22.7%). Learning aids used in different units included lectures (320, 68%), videos (269, 57%), simulation training (151, 32%), and cadavers (66, 14%).

A total of 372 (79%) respondents felt that 10–30 cases were required to become proficient, and 10–30 cases were required to maintain competency. Furthermore, 336 (71%) felt that trainees should be competent in performing vaginal hysterectomy prior to completing general training in obstetrics and gynaecology.

## 4 Discussion

### 4.1 Main Findings

Our survey included primarily urogynaecologists, revealing that despite the vast majority (63%) preferring the removal of the uterus in the presence of apical descent, two‐thirds perform fewer than 30 vaginal hysterectomies annually, and 45% noted a reduction in numbers. Additionally, 71% felt that vaginal hysterectomy is an essential part of training and that trainees should be competent in performing this procedure prior to completing general training in obstetrics and gynaecology.

These findings challenge the assumption that the majority of practitioners today prefer uterine‐preserving procedures and raise real concerns about the future of vaginal hysterectomy training in light of declining numbers. The preference demonstrated in our study for uterine removal contradicts the trend towards uterine‐sparing surgeries [4, 6], highlighting a potential gap between preferred practices and actual surgical training and experience. The dilemma lies in balancing the preference for minimally invasive procedures with the necessity of maintaining proficiency in traditional surgical techniques like vaginal hysterectomy [13].

### 4.2 Strengths and Limitations

This study provides valuable insights into the current practices and training needs of urogynaecologists. The large sample size and focus on a specialised population highlight significant trends and preferences that can inform future training programs and policy decisions. Several limitations must be acknowledged. First, the study surveyed members of IUGA and EUGA, which may not represent the broader gynaecologist population, introducing selection bias. This could lead to an overrepresentation of views specific to urogynaecology, limiting the generalisability of the findings. Additionally, we do not have exact numbers of members who received the survey but declined to respond, further contributing to selection bias. Future studies could mitigate this by tracking response rates more accurately and conducting follow‐up with non‐responders. Second, the questionnaire was not validated, potentially affecting the reliability of the responses. Third, recall and response bias are possible, as respondents may not accurately remember or report their practices. This could skew the results, either overestimating or underestimating certain trends.

Despite these limitations, the findings offer valuable insights into the evolving landscape of gynaecological surgery and highlight the need for comprehensive training. Future research could benefit from using validated surveys and methods to minimise recall bias, such as cross‐referencing with surgical records.

### 4.3 Interpretation

The study underscores significant concerns about the training of future gynaecologists. The low volume of vaginal hysterectomies performed by specialists could impact the proficiency of trainees. Published literature supports the idea that surgical volume is critical for achieving and maintaining proficiency [10]. Studies have shown that high‐volume surgeons have better patient outcomes compared to low‐volume surgeons [9]. Using validated vaginal surgery skills assessment tools, it was found that it takes, on average, 27 vaginal hysterectomies to achieve minimal competency for this procedure [14]. Most respondents in our survey were even more liberal and felt it took as few as 10–30 cases to achieve proficiency. Despite evidence‐based medicine [14] and expert opinions provided in our survey, training requirements for general obstetrics and gynaecology trainees in the US and UK are much lower, between 5 and 15 cases over several years [15].

This discrepancy is further supported by evidence in the literature, indicating that operative experience has appeared to decline in recent years due to an increase in the number of trainees coupled with a decrease in working hours due to changes in work policies [16]. In a study conducted over an 8‐year period in Ireland (2014–2021) [17], an overall decrease in both trainees' and trainers' confidence was demonstrated. This is particularly true for major gynaecological procedures; specifically, between the years 2014–2021, there was a 50% shortfall in senior trainees' ability to perform vaginal hysterectomy. Furthermore, despite the fact that most respondents in our survey believe trainees should be proficient in performing vaginal hysterectomy before completing general obstetrics and gynaecology training, this is apparently not the situation. A recent study from Canada [18] highlights that this expectation is not consistently being met, as many trainees may not achieve this competency due to limited exposure and surgical opportunities to perform the procedure.

These findings support the observation that the proportion of general obstetric and gynaecology graduates accepted into fellowships rise from 7% in 2000 to 19.5% in 2012 [19]. This drive to sub‐specialisation is likely in response to the lack of surgical experience during general training. As more gynaecologists move into sub‐specialties, the question arises as to who will perform less complex cases if generalists are not adequately trained.

When comparing these findings with other surgical specialties, similar concerns regarding declining operative experience are evident. In general surgery, the reduction in work hours due to duty hour restrictions has led to decreased exposure to certain procedures, particularly in complex cases, thereby impacting trainees' proficiency [20], which has raised concerns about their readiness for independent practice. Similarly, in vascular surgery [21], there has been a shift towards endovascular procedures, leading to reduced exposure to open surgical techniques. In spinal surgery [22], training trends within neurological and orthopaedic surgery residency programs have shown similar challenges, with fluctuations in case volumes and a growing reliance on advanced technologies potentially affecting hands‐on experience. Collectively, these trends across multiple disciplines underscore a broader issue; ensuring that surgical trainees receive adequate exposure and training to achieve and maintain proficiency in essential procedures.

Given these challenges, the incorporation of simulation and other training models has become particularly valuable in gynaecology, where the hands‐on experience for complex procedures like vaginal hysterectomy is increasingly limited [23]. Simulation‐based training not only enhances technical skills but also improves decision‐making and confidence in performing procedures [23]. Studies [24, 25, 26] have demonstrated the effectiveness of simulation in surgical training across various specialties, including gynaecology, general surgery, and vascular surgery. By integrating these methods into gynaecology training programs, educators can ensure that trainees receive the comprehensive education needed to maintain proficiency in both traditional and minimally invasive techniques. Furthermore, these training models offer a standardised assessment of competence, which is crucial for ensuring that all trainees achieve the required level of proficiency before entering independent practice.

To address the evolving needs in gynaecological surgery, it is crucial to ensure that future gynaecologists receive comprehensive training in both traditional procedures like vaginal hysterectomy and emerging minimally invasive techniques. Ensuring safety in surgical practice is paramount, particularly as procedure numbers decline. Without addressing these training gaps, safety risks will inevitably increase. By enhancing surgical education and addressing current training deficits, the medical community can better prepare future surgeons to deliver optimal and safe care to their patients, thus safeguarding the quality of gynaecological practice in the face of changing surgical trends.

## 5 Conclusion

Vaginal hysterectomy is far from obsolete; it remains vital for managing POP. However, multiple factors, including surgical training, influence the decision‐making process. The potential decline in the number of procedures performed by specialists risks the quality of training for future gynaecologists, necessitating a re‐evaluation of training requirements and practices to ensure essential skills are preserved and adequately taught. This survey highlights the need for a comprehensive approach in surgical training, ensuring future gynaecologists are proficient in all available surgical techniques. Addressing these training gaps will better prepare the next generation of surgeons to meet the diverse needs of their patients.

## Author Contributions

R.R. conceptualisation, project administration, methodology, investigation, formal analysis, manuscript writing, and editing. M.O.C. data curation, project administration, editing. C.M.M. investigation, manuscript writing, and editing. B.A.O. writing – review & editing. Y.D. project administration, writing & editing. O.E.O. conceptualisation, methodology, investigation, writing – review & editing. All authors agree with the final version of the manuscript and its submission to journal.

## Ethics Statement

This questionnaire was distributed in accordance with the regulations established by the International Urogynecological Association (IUGA) and the European Urogynaecological Association (EUGA) executive boards for medical practitioners, with an approved waiver from the Helsinki Ethics Committee.

## Conflicts of Interest

The authors declare no conflicts of interest.

## Supporting information


Appendix S1.

## Data Availability

Data will be made available from the corresponding author upon reasonable request.
