# Supplementary material for: Preserving Essential Skills: The Future of Vaginal Hysterectomy Training in Urogynaecology
Source: BJOG. 2024 Oct 2;132(2):205–11. doi: 10.1111/1471-0528.17974 (PMC11625650; doi:10.1111/1471-0528.17974)
Supplement: Supplementary file 1 — Appendix S1. [file BJO-132-205-s001.pdf]

Thank you for taking the time to complete this questionnaire on the management of apical prolapse and in particular operator preferences and training with regard to vaginal hysterectomy. This survey should take between 5-10 minutes to complete.

* 1. What is your specialty?

- ◯ Uroynaecology
- ◯ Gynaecology
- ◯ Obstetrician and Gynaecologist
- ◯ Urology
- ◯ Obstetrician
- ◯ Physician
- ◯ Surgeon
- ◯ Nursing
- ◯ Physiotherapy
- ◯ Allied Healthcare professional

Other (please specify)

* 2. What position do you currently hold?

- ◯ Consultant
- ◯ Staff grade
- ◯ Fellow
- ◯ Resident/Registrar
- ◯ Other

Other (please specify)

* 3. Which best describes the institution you work in?

- ◯ University Affiliated hospital
- ◯ Private hospital
- ◯ Tertiary referral centre
- ◯ Academic centre
- ◯ District hospital

* 4. How many years have you been in practice?

- 1-5
- 5-10
- 10-15
- 15-20
- >20

* 5. What percentage of your times is spent on Urogynaecology exclusively?

- 0-10%
- 11-25%
- 26-50%
- 51-75%
- >75%

* 6. Have you completed a Fellowship?

- Yes
- No

7. What was the Fellowship in?

- Urogynaecology and Pelvic reconstructive surgery
- Female Urology
- Robotic Surgery
- Minimally invasive Surgery
- Other (please specify)

8. If yes, how many years was your Fellowship for?

9. Where did you do your Fellowship?

- Mainland Europe
- British Isles
- Australia/ New Zealand
- United States of America
- Other (please specify)

* 10. In women with uterine prolapse, Which is your preference

- ◯ Uterine preservation
- ◯ Uterine removal

* 11. What factors influence your decision

- ☐ Patient preference
- ☐ Patient age
- ☐ Post menopause
- ☐ Surgical preference/expertise
- ☐ Surgical training
- ☐ Prolapse score
- ☐ Related symptoms
- ☐ Co morbidities
- ☐ Recurrence rates
- ☐ Renumeration rates
- ☐ Availability of surgical equipment
- ☐ Patient BMI
- ☐ Ongoing hospital based clinical research projects

* 12. Which of these procedure are you proficient in?

- ☐ Vaginal hysterectomy and native tissue repair
- ☐ Vaginal hysterectomy and Mesh kit repair
- ☐ Vaginal sacrospinous hysteropexy
- ☐ Vaginal uterosacral plication
- ☐ Open Abdominal sacrohysteropexy
- ☐ Abdominal uterosacral plication
- ☐ Abdominal sacrocervicopexy with concomitant supracervical hysterectomy
- ☐ Laparoscopic Abdominal sacrohysteropexy
- ☐ Robot- assisted Abdominal sacrohysteropexy
- ☐ Laparoscopic uterosacral plication
- ☐ Robot-assisted uterosacral plication
- ☐ Laparoscopic sacrocervicopexy with concomitant supracervical hysterectomy
- ☐ Robot-assisted sacrocervicopexy with concomitant supracervical hysterectomy

* 13. Which of these procedures do you perform more than 10 times per year

- [ ] Vaginal hysterectomy and native tissue repair
- [ ] Vaginal hysterectomy and Mesh kit repair
- [ ] Vaginal sacrospinous hysteropexy
- [ ] Vaginal uterosacral plication
- [ ] Open Abdominal sacrohysteropexy
- [ ] Abdominal uterosacral plication
- [ ] Abdominal sacrocervicopexy with concomitant supracervical hysterectomy
- [ ] Laparoscopic Abdominal sacrohysteropexy
- [ ] Robot- assisted Abdominal sacrohysteropexy
- [ ] Laparoscopic uterosacral plication
- [ ] Robot-assisted uterosacral plication
- [ ] Laparoscopic sacrocervicopexy with concomitant supracervical hysterectomy
- [ ] Robot-assisted sacrocervicopexy with concomitant supracervical hysterectomy

* 14. Do you perform Vaginal hysterectomy ?

- ○ Yes
- ○ No

* 15. About how many vaginal hysterectomies have you performed in the past 12 months?

- ○ <10
- ○ 11-30
- ○ 31-50
- ○ >50

* 16. How has the number of your vaginal hysterectomy procedures changed compared to 5-10 years ago?

- ○ Increasing
- ○ Decreasing
- ○ Staying the same

* 17. Do you routinely perform a BSO at the time of vaginal hysterectomy

- [ ] yes in all women
- [ ] yes in all postmenopausal women
- [ ] yes only in women with ovarian pathology
- [ ] yes in women who request it
- [ ] no

* 18. Prior to clamping the uterosacral ligaments do you usually

- ◯ enter the peritoneal cavity posteriorly only
- ◯ enter the peritoneal cavity anteriorly and posteriorly
- ◯ clamp the uterosacral ligaments prior to entering the peritoneal cavity

* 19. Do you routinely use a suspensory technique for the vaginal vault

- ◯ Yes
- ◯ No

* 20. If yes, which technique do you routinely use

- ◯ Mc Calls culdoplasty
- ◯ Modified Mc Calls culdoplasty
- ◯ Uterosacral suspensory suture
- ◯ Sacrospinous fixation
- ◯ Other (please specify)

* 21. If performing concomitant pelvic floor repair, do you routinely use a native tissue repair

- ◯ Yes
- ◯ No

22. If not, what material do you use for the repair

- ◯ Polypropylene mesh
- ◯ Bovine graft
- ◯ Porcine graft
- ◯ Biologic coated polyprolene mesh
- ◯ Other (please specify)

* 23. At vaginal hysterectomy for POP, do you routinely perform an anti-incontinence procedure

- ◯ Yes
- ◯ No

24. If yes, what procedure do you use

- Mid-urethral sling
- Burch colposuspension
- Marshall-Marchetti-Krantz
- Other (please specify)

* 25. Do you routinely perform cystoscopy post vaginal hysterectomy

- Yes
- No

* 26. With regard to intraoperative complications during vaginal hysterectomy. How do you manage a bladder injury?

- Close the defect and continue the procedure
- Close the defect, assess ureters and continue the procedure
- Close the defect and abandon the surgery
- Call Urology to close the defect and then continue the surgery
- Call Urology to close the defect and abandon the procedure

* 27. With regard to intraoperative complications during vaginal hysterectomy. How do you manage a rectal injury ?

- Close the defect and continue the procedure
- Close the defect and abandon the procedure
- Call the colorectal team to close the defect and continue the procedure
- Call the colorectal team to close the defect and abandon the procedure

* 28. Learning the technique of vaginal hysterectomy, how difficult would you describe each of the following steps as being ?

	very easy	easy	moderate	difficult	very difficult
Colpotomy	○	○	○	○	○
Opening the pouch of Douglas	○	○	○	○	○
Opening the uterovesical fold	○	○	○	○	○
Clamping and cutting the uterosacral ligaments	○	○	○	○	○
Suture ligating the uterosacral ligament	○	○	○	○	○
Clamping , cutting and suturing the uterine vessels and round ligament	○	○	○	○	○
Performing a heaney suture	○	○	○	○	○
Clamping, cutting and suturing the infundibulopelvic ligament	○	○	○	○	○
Performing the suspensory suture	○	○	○	○	○
Closing the vault	○	○	○	○	○

* 29. What teaching tools are used in your department to aide teaching trainees how to perform surgery?

- [ ] Didactic lectures
- [ ] Video aides
- [ ] Simulation training
- [ ] Wet lab
- [ ] Cadaveric work shops
- [ ] Other (please specify)

* 30. To gain competency what should be the minimum number of vaginal hysterectomies performed by a trainee during training

- [ ] <10
- [ ] 10-20
- [ ] 20-30
- [ ] >30

* 31. To maintain competency how many vaginal hysterectomies should a gynaecologist perform per year

- ☐ <10
- ☐ 10-20
- ☐ 20-30
- ☐ >30

* 32. Do you think trainees should be able to perform a vaginal hysterectomy independently on completion of their general training in Obstetrics and Gynaecology?

- ☐ Yes
- ☐ No

33. We would grateful for any feedback or comments relating to vaginal hysterectomies

Thank you for taking the time to complete this questionnaire.
